# Health Technology Readiness amongst Patients with Suspected Breast Cancer Using the READHY-tool - a Cross-sectional Study

**DOI:** 10.1007/s10916-023-02016-0

**Published:** 2023-11-16

**Authors:** Martin Sollie, Marianne Hansen, Jørn Bo Thomsen

**Affiliations:** 1 Research Unit for Plastic Surgery, Odense University Hospital, J.B. Winsløws Vej 4, Indgang 20 Penthouse 2. sal, Odense C, DK 5000 Denmark; 2 OPEN, Open Patient Data Explorative Network, Odense University Hospital, Region of Southern, Odense, Denmark; 3 Department of Plastic Surgery, Odense University Hospital, J.B. Winsløws Vej 4, Indgang 20 Penthouse 2. sal, Odense C, DK 5000 Denmark

**Keywords:** Breast cancer, Health technology readiness, Health technology

## Abstract

Information technologies are increasingly used when informing patients about their disease, treatment and prognosis. These digital platforms have many advantages compared to traditional education interventions. However, there are concerns that some patients may have difficulty with this mode of information delivery. Newly diagnosed breast cancer patients are dependent on understanding their treatment options to make informed treatment decisions. Yet, there is a lack of published material on breast cancer patients and their relationship with technology. We aimed to assess health technology readiness profiles amongst women with a suspected breast cancer diagnosis. Secondly, we wanted to investigate the potential differences between these profiles according to sociodemographic factors and the patients´ current use of technology. This cross-sectional study used the Readiness and Enablement Index for Health Technology (READHY) questionnaire. We included all patients (n = 92) referred to our department with suspected breast cancer. Cluster analysis revealed three distinct profiles: medium (n = 54), high (n = 18), and low (n = 20) health technology readiness. The third profile showed difficulties in health literacy, eHealth literacy, and health insights, along with higher emotional stress. Our study found that most patients had medium to high health technology readiness, but we also identified a group with lower health technology readiness. Based on our results, healthcare personnel dealing with women with suspected breast cancer should be aware of patients struggling with health technology. Age and technology familiarity may indicate vulnerable patients. Future studies should explore optimal methods for information delivery to these distinct profiles and evaluate the long-term impacts.

## Introduction

Being diagnosed with breast cancer can negatively impact mental health, with studies showing higher anxiety levels, depressive symptoms, distress and trauma-related symptoms [1]. Following initial diagnosis, the patients often undergo further diagnostics and treatment [2]. These steps are usually initiated and performed as fast as possible to avoid delay in treatment. Patients, in collaboration with healthcare professionals, make informed treatment decisions. To facilitate this, patients must receive relevant and comprehensive information to choose their preferred treatment.

Previously, this information was mainly given orally in consultations with healthcare personnel or using printed materials. Now, in the technology era, the use of digital solutions for patient information is rapidly increasing, and a growing body of evidence supports the use of digital health technology [3, 4]. These platforms have many advantages, such as being more time- and cost-effective than traditional education interventions. It also enables us to provide relevant information uniformly to all our patients [5]. Though the use of digital information is increasing, we have yet to investigate whether patients are ready for this change thoroughly. To better facilitate patients handling their disease, we must understand their information needs [6]. Published studies on other populations, such as those with other types of cancer, inflammatory bowel disease or diabetes type II, have revealed significant inter-population differences in the use and approach to information technology. Some of the proven factors that influence the use of information technology are emotional distress, familiarity with technology and age [7, 8].

Measuring digital health technology readiness can be challenging, and several questionnaires have been designed for this purpose. One challenge today is the offer of many smaller questionnaires with different comprehensibility and areas of interest [9]. There is, therefore, a need for more reliable measuring tools [9]. One way to evaluate the health technology readiness of our patients is the newly developed Multidimensional Readiness and Enablement Index for Health Technology (READHY) questionnaire [10]. The tool consists of several questionnaires and assesses the many aspects of health technology readiness, such as technology skills, emotional distress, social support and ability to engage with digital services. It can also identify subpopulations within patient groups with problems using and understanding health technology [10].

There is limited research available on the readiness to use health technology in women newly diagnosed or suspected of having breast cancer. To address this, we´ve initiated the first prospective study utilising the READHY tool for this patient group.

The aims of this study are (1) to identify health technology readiness profiles amongst women with a suspected breast cancer diagnosis. (2) to investigate the differences between these profiles according to sociodemographic factors and their current use of technology.

## Methods

We designed this study as a cross-sectional study using the READHY questionnaire.

It was registered at ClinicalTrials.gov before initiation. Identifier: NCT04745117 [11]. The study was reported per The Strengthening the Reporting of Observational Studies in Epidemiology (STROBE) Statement [12].

### Setting and Participants

The study was conducted at the Department of Plastic Surgery at Odense University Hospital. The participants were constituted by a convenience sample obtained from 01.03.2021 through 31.12.2021. All patients referred to us with suspected breast cancer were asked to participate. Our department receives patients from the area of Funen and the surrounding islands. The exclusion criteria were: not being able to understand and read Danish.

Patients were referred via the official screening program for breast cancer in Denmark, where all women between 50 - and 69 years are offered a mammogram every two years. If the clinical mammogram raised suspicion of breast cancer, they were referred to us as the closest breast surgery centre. Additionally, all patients of all ages with clinical suspicion of breast cancer were referred by general practitioners. We asked the patients to complete the questionnaire on their first or second visit to our department.

The patients answered the questionnaires electronically using a tablet. If the patients wanted to fill out the questionnaires on paper, this was arranged by the attending nurse. The patients were informed orally and in writing about the study before giving consent to participate. The questionnaire constituted a section of demographic data and the READHY tool.

We collected the following demographic data: gender, age, the highest level of education, cohabitation status, and source of income. We also asked the participants if they owned a smartwatch, tablet, or computer and their primary purpose when using information technology (IT).

### The Readiness and Enablement Index for Health Technology (READHY)

**Fig. 1:**
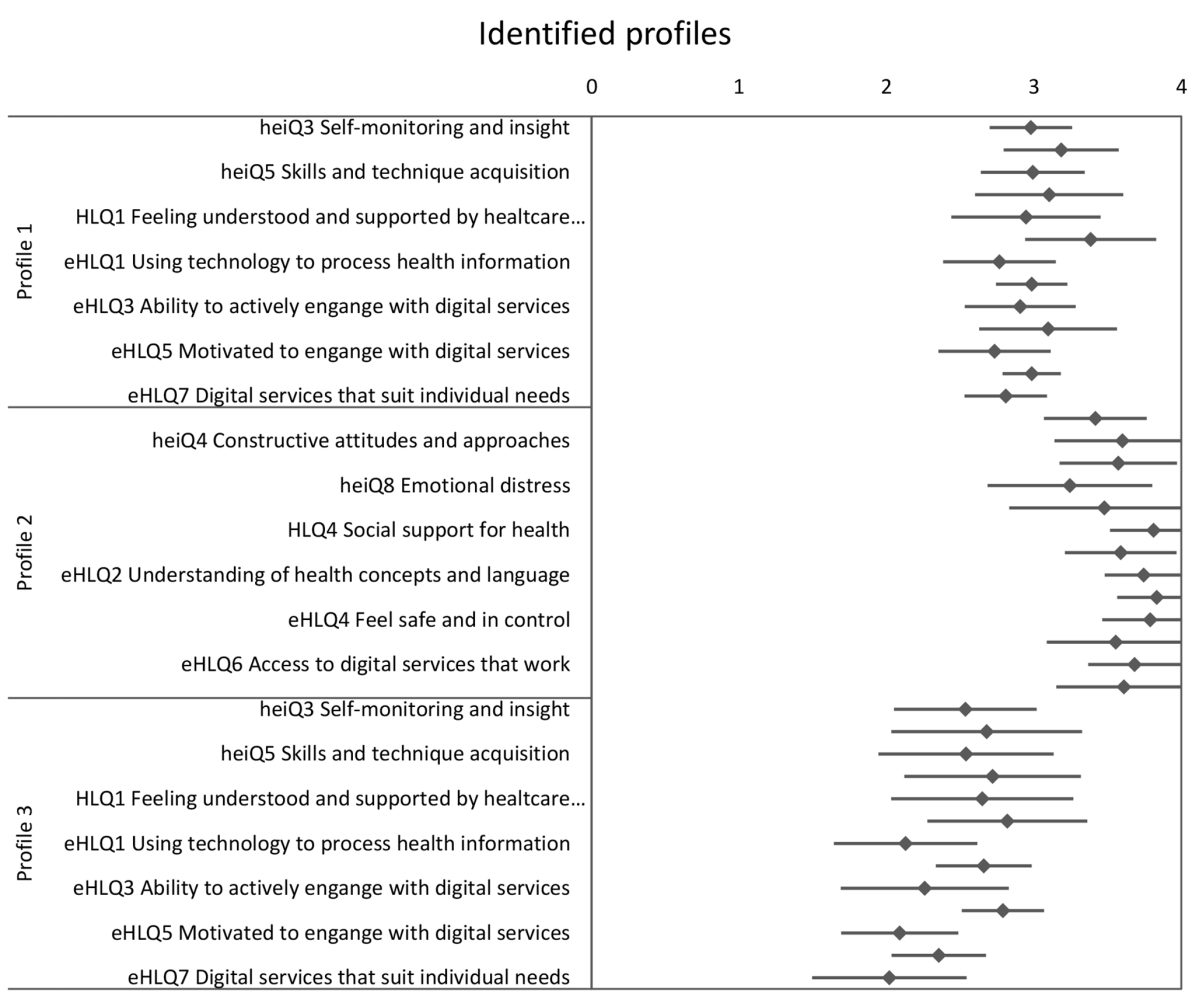
Readiness and Enablement Index for Health Technology (READHY) scale scores for the three identified profiles based on cluster analysis. heiQ: Health Education Impact Questionnaire; HLQ: Health Literacy Questionnaire; eHLQ: eHealth Literacy Questionnaire. Data are presented as mean (SD). heiQ8 was reversed (i.e., a high score indicated a low level of emotional distress)

The READHY tool was used to assess readiness for health technology. The tool consists of the eHealth Literacy Questionnaire (eHLQ) [13], which includes seven scales, supplemented with four scales from the Health Education Impact Questionnaire (heiQ) [14] and two scales from the Health Literacy Questionnaire (HLQ) [15]. The individual scales and associated questionnaires can be found in Fig. 1. Together, these scales capture eHealth literacy, self-management, and social context. The READHY tool is a validated assessment tool [10]. The 13 scales were assessed using 65 items. Each item was presented to the participant as a statement and scored on a 4-point rating scale, ranging from 1 = strongly disagree to 4 = strongly agree. The overall score of each scale was calculated as the mean score of the 4–6 items (i.e., statements) that constitute the scale. If less than 50% of items in a scale were answered, the scale was regarded as missing; in those cases, the survey was considered incomplete.

### Statistical Methods

We consulted a clinical statistician before initiating the study. Based on previously published data using the READHY tool, we were advised to aim for approximately one hundred participants.

Our data-analysis method was based on a previously published study with a similar aim by Thorsen et al. [8]. We used a data-driven approach with a combination of hierarchical and K-means cluster analysis to divide participants into clusters depending on their level of readiness for health technology. We have chosen to refer to these clusters as profiles.

We used the two-step k-means analysis and Bonferroni post hoc analyses using the one-way analysis of variance (ANOVA) to decide the optimal number of clusters. The results assessed three profiles as the best fit and two as the second best fit. We conducted K-means cluster analyses for three profiles in 8 iterations. The difference between profiles for each ready scale was assessed using a one-way ANOVA.

For sociodemographic and IT-related data, the differences between the profiles were tested using the Fisher exact test for frequencies and the one-way ANOVA for continuous variables. Frequencies are reported as numbers and proportions, and continuous variables are reported as mean and standard deviation (SD).

Data were analysed as observed. No imputations were used to replace missing data.

Data were analysed using SPSS version 28 [16]. The statistical analyses were performed under the guidance of a clinical statistician.

## Results

### Readiness for Health Technology

**Fig. 2:**
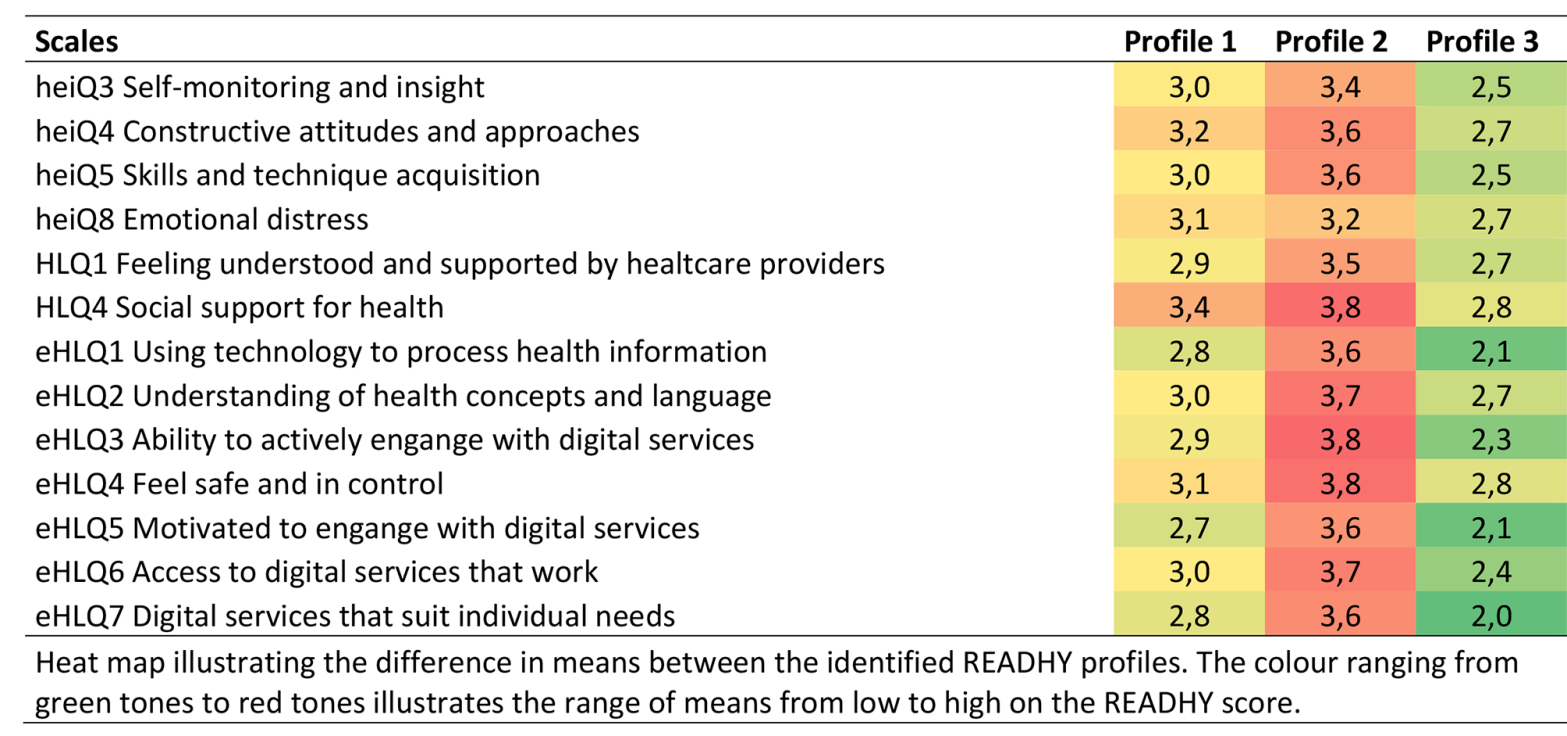
Heat map illustrating the difference in means between the identified READHY profiles. The colour ranging from green to red tones demonstrates the range of means from low to high on the READHY score

The cluster analyses of the total READHY scores resulted in three distinct profiles of participants. They are presented in Figs. 1 and 2. The different profiles scored high, medium and low in their overall READHY score. Profile 2 (n = 18) consistently scored high on all scales, while profile 3 (20) revealed low scores. Profile 1 (n = 54) consistently scored medium on all scales. 60% of participants were allocated to profile 1. The other two profiles, 2 and 3, comprised 20% of the participants each.

Profile 3 scored lowest on all parameters when assessing the READHY items regarding health literacy, the eHLQ and HLQ, indicating that these patients felt less supported by their healthcare providers and social network. They also reported low motivation to engage with digital services and insufficient access to digital services that suit their needs.

The same pattern was seen in the heiQ, where profile 3 also consistently scores the lowest, indicating problems with self-monitoring and insight, constructive attitudes and approaches to health education. They also reported a higher emotional stress level than the other two profiles.

### Sociodemographic Characteristics

The sociodemographic characteristics of the total population and the individual profiles are presented in Table 1. When comparing the different profiles, we observed that participants in profile 2, with the highest scores, were younger (57.9 y) than the participants in profiles 1 (62.5 y) and 3 (69.5 y) (P = 0.013). We did not detect any statistically significant differences in the groups regarding their highest level of education, cohabitation status or source of income. Profile 3 had the highest portion of patients, with only comprehensive school as their highest level of education. Yet, the frequency differences were not statistically significantly different from the other two profiles. Regarding cohabitation status, there was a tendency for patients in profile 3 more often lived alone compared to the other two groups, but no statistically significant difference was detected. Patients in profile 3 had the lowest frequency of salary as income, with a higher portion receiving retirement pension or public income support.

**Table 1:** Sociodemographic characteristics and access to technology of participants (N = 92) across the identified profiles. Data are presented as mean (SD) for continuous variables and number (proportions) for frequencies

Characterstics	Total (N = 92)	Profile 1 (N = 54)	Profile 2 (N = 18)	Profile 3 (N = 20)	P-value
**Age, mean (SD)**	63.1 (12.4)	62.5 (11.9)	57.9 (12.7) c	69.5 (11.1) b	*0.013*
**Highest attained level of education, n (%)**					*0.277*
Comprehensive school	16 (17.4)	8 (14.8)	1 (5.6)	7 (35.0)	
Short education (2–3 y)	40 (43.5)	25 (46.3)	8 (44.4)	7 (35.0)	
Medium education (3–4 y)	34 (37.0)	20 (37.0)	8 (44.4)	6 (30.0)	
Long education (4 + y)	2 (2.1)	1 (1.9)	1 (5.6)	0 (0.0)	
**Cohabitation status, n (%)**					*0.451*
Living alone	23 (25.0)	13 (24.1)	3 (16.7)	7 (35.0)	
Living with spouse and/or children	69 (69.0)	41 (75.9)	15 (83.3)	13 (65.0)	
**Source of income**					*0.650*
Salary	38 (41.3)	25 (46.3)	10 (55.6)	3 (15.0)	
Retirement pension	11 (11.9)	6 (11.1)	1 (5.6)	4 (20.0)	
Public income support/ no incomes	43 (46.7)	23 (42.6)	7 (38.8)	13 (65.0)	
**Do you own any of these IT aids?**					
Smartwatch?					*0.845*
YES	13 (14.3)	8 (14.8)	3 (16.7)	2 (10.0)	
NO	79 (85.8)	46 (85.2)	15 (83.3)	18 (90.0)	
Smartphone?					*0.006*
YES	85 (92.3)	53 (98.1) c	17 (94.4)	15 (75.0) a	
NO	7 (7.6)	1 (1.9) c	1 (5.6)	5 (25.0) a	
Computer?					*0.130*
YES	77 (83.7)	46 (85.2)	17 (94.4)	14 (70.0)	
NO	15 (16.3)	8 (14.8)	1 (5.6)	6 (30.0)	
Tablet?					*0.030*
YES	64 (69.5)	40 (74.1) c	16 (88.8) c	8 (40.0) a,b	
NO	28 (30.4)	14 (25.9) c	2 (11.1) c	12 (60.0) a,b	
**How do you use technology in your daily life?**					
Excersize?					*0.006*
YES	17 (18.5)	8 (14.8) b	8 (44.4) a,c	1 (5.0) b	
NO	75 (8,1)	46 (85.2) b	10 (55.5) a,c	19 (95.0) b	
Work?					*0.005*
YES	38 (41.3)	23 (42.6) c	12 (66.7) c	3 (15.0) a,b	
NO	54 (58.7)	31 (57.4) c	6 (33.3) c	17 (85.0) a,b	
Seeking information?					*0.009*
YES	76 (82,6)	47 (92.2) c	17 (94.4) c	12 (60.0) a,b	
NO	16 (17.4)	7 (13.7) c	1 (5.6) c	8 (40.0) a,b	
Communication?					*0.496*
YES	77 (83.7)	46 (85.2)	16 (88.9)	15 (75.0)	
NO	15 (16.3)	8 (14.8)	2 (11.1)	5 (25.0)	
Entertainment?					*< 0.001*
YES	62 (67.4)	39 (72.2) c	17 (94.4) c	6 (30.0) a,b	
NO	30 (32.6)	15 (27.8) c	1 (5.6) c	14 (70.0) a,b	

a) Different than profile 1

b) Different than profile 2

c) Different than profile 3

### IT use

The reported IT use is presented in Table 1. The participants in the lowest scoring, profile 3, had a statistically significant lower percentage of smartphone ownership (75%) compared to profiles 1 (98%) and 2 (94%) (p = 0.006). The same was observed regarding tablet ownership, with a rate of 40% in profile 3 and 74% and 89% in profiles 1 and 2, respectively (p = 0.03). We did not detect any significant difference in computer and smartwatch ownership frequencies.

When asked about their use of IT in daily life, the youngest group, profile 2, had the highest use of technology during fitness and exercise (15%), compared to the two other profiles, 1 (15%) and 3 (5%). The difference was statistically significant across the profiles (p = 0.006). Similar results were observed for the variable “IT in work situations”. When asked about their use of IT in seeking information, almost all participants in profiles 1 and 2 stated yes, while the frequency was much lower in profile 3 (92.2%, 94.4% and 60.0%, respectively. p = 0.009). For communication purposes, there was no statistically significant difference across the groups. Profile 2 used the most IT for entertainment purposes (94.4%), while profile 1 (72.2%) and profile 3 (30.0%) stated much lower usage (P = < 0.001).

## Discussion

This is the first study investigating the health technology readiness amongst patients referred to the hospital with suspected breast cancer using the READHY tool.

Most patients, approximately 80%, demonstrated medium to high levels of health technology readiness. Our study’s main finding is identifying patients with lower READHY scores in all the measured parameters. This group comprised approximately 20% of the total participants in our sample. This group had the lowest scores on the Health Education Impact Questionnaire (heiQ), indicating a higher level of emotional stress, less self-monitoring and health insight, and a lower skill set to manage their health. The same pattern was seen in the eHealth Literacy Questionnaire (HLQ), with more issues related to locating, using, and understanding digital healthcare information. They also scored the lowest on The Health Literacy Questionnaire (HLQ) when asked about the support system surrounding their health, their ability to take action regarding their healthcare, and their understanding of information about their health. This aligns with other studies identifying subpopulations of lower health technology readiness in other patient groups, such as diabetes type II and irritable bowel disease [7, 8]. Factors associated with these subpopulations were, amongst others, age, emotional well-being, familiarity with IT and degree of eHealth literacy, all in line with our study [8]. The authors have not identified other studies on cancer populations´ health technology readiness using the READHY tool.

We investigated sociodemographic factors and IT use to find some factors that could help us locate these potentially more vulnerable patients. The only sociodemographic factor associated with profile 3 was age, as these patients were statistically significantly older than the other two. We also observed that patients in profile 3 were less likely to be familiar with using technology for exercise, seeking information and entertainment purposes. This is not surprising as previous studies have found that older patients may be unable to or wish to engage with electronic health resources [17, 18].

### Limitations

The main limitation of this study is the cross-sectional design, with a lack of follow-up. We have no data on how the patients eventually received and understood the information given to them. We did not register the diagnostics journey of our patient before being referred to our department, and there is a risk that being referred from the screening program or via the general practitioner could influence the READHY results, especially regarding the items of emotional distress. Our convenience sample constituted only ninety-two patients, which could be low when making a cluster analysis. We do, however, feel that the results clearly indicate a substantial sized, more vulnerable group of patients within this patient category.

Some of the questions in the questionnaire may also not be appropriate for the patients in profile 3. Specifically with regards to the questions on the use of technology in daily life, the answers to the question “Do you use technology for work?” may be misleading, as the mean age of participants was 69.5 years old and above the average retirement age, compared to the other two groups.

We aimed to invite all patients referred to our department during the study period to participate in our study. We do not have the exact number of patients declining to participate in our survey. Due to oversight, we cannot rule out that eligible patients have not been asked to participate. This lack of information on the demographics of these patients is, therefore, a potential weakness of this study.

The future impact of these results is difficult to predict. In our population, 20% of patients were allocated to profile 3, which scored the lowest in health technology readiness. These patients were statistically significantly older than patients in the other two groups, and we found that they less often owned a tablet or a smartphone. We have not, however, identified a specific cut-off point regarding age and low scores on the READHY that would enable us to identify patients who may need additional or alternative information.

## Conclusion

Our study found that most patients with suspected breast cancer had medium to high health technology readiness. We also identified a group with lower health technology readiness. Based on our results, we advise healthcare personnel dealing with women with suspected breast cancer to be aware of patients struggling with health technology. Age and technology familiarity may indicate vulnerable patients. More studies investigating different modes of information delivery with follow-ups are needed.

## Data Availability

The datasets generated and/or analysed during the current study are not publicly available due to the Danish Data Protection laws. Further inquiries can be directed to the corresponding author.
